# Efficient Conversion of Pine Wood Lignin to Phenol

**DOI:** 10.1002/cssc.202000485

**Published:** 2020-03-10

**Authors:** Xianhong Ouyang, Xiaoming Huang, Michael D. Boot, Emiel J. M. Hensen

**Affiliations:** ^1^ Laboratory of Inorganic Materials and Catalysis Department of Chemical Engineering and Chemistry Eindhoven University of Technology P.O. Box 513, 5600 MB Eindhoven The Netherlands; ^2^ Energy Technology Department of Mechanical Engineering Eindhoven University of Technology P.O. Box 513, 5600 MB Eindhoven The Netherlands; ^3^ Current address: Polymer Technology Group Eindhoven (PTG/e) B.V. P.O. Box 6284 5600 HG Eindhoven The Netherlands

**Keywords:** biomass, demethoxylation, lignin-first, phenol, transalkylation

## Abstract

Obtaining chemical building blocks from biomass is attractive for meeting sustainability targets. Herein, an effective approach was developed to convert the lignin part of woody biomass into phenol, which is a valuable base chemical. Monomeric alkylmethoxyphenols were obtained from pinewood, rich in guaiacol‐type lignin, through Pt/C‐catalyzed reductive depolymerization. In a second step, an optimized MoP/SiO_2_ catalyst was used to selectively remove methoxy groups in these lignin monomers to generate 4‐alkylphenols, which were then dealkylated by zeolite‐catalyzed transalkylation to a benzene stream. The overall yield of phenol based on the initial lignin content in pinewood was 9.6 mol %.

## Graphical abstract

**Into the woods**: Phenol, a valuable base chemical, is obtained from renewable woody biomass through lignin reductive depolymerization, demethoxylation, and transalkylation. The promising overall yield of phenol based on the initial lignin content in pinewood is 9.6 mol %

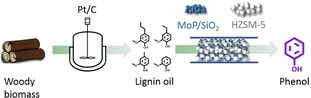

Lignocellulosic biomass holds great promise as a source of sustainable chemicals replacing fossil resources. Lignin is the largest renewable source of aromatics, which explains the substantial interest from the chemical industry.^1^ Many different methods have already been explored to depolymerize the recalcitrant polyphenolic network of lignin into aromatic monomers.^2^ Among these, the lignin‐first process (LFP) stands out in terms of the high yield of monomers (in the form of alkylmethoxyphenols), using optimized catalysts to cleave the linkages that bind lignin fragments to hemicellulose and lignin intralinkages.^3^ Because the market demand for these alkylmethoxyphenols is relatively small, it is desirable to convert them into bulk chemical building blocks.^4^ For instance, Song et al. described an approach to convert 2‐methoxy‐4‐propylphenol to terephthalic acid through demethoxylation, carbonylation, and oxidation.^5^ Other than terephthalic acid, the removal of the methoxy functionalities and alkyl side groups can give access to phenol, which is a valuable intermediate in the manufacture of agrochemicals, detergents, plastics, pharmaceuticals, dyes, and plasticizers.^6^ We earlier explored a combination of hydrodemethoxylation (HDMeO) and transalkylation of the alkyl groups to a benzene co‐reactant to obtain phenol from the same lignin model compound in a one‐step approach.^7^ Full conversion of 2‐methoxy‐4‐propylphenol, representative for guaiacol‐type alkylmethoxylphenols obtained from LFP, was achieved with a reasonable phenol yield of 60 %. The bifunctional catalytic approach combined HDMeO catalyzed by Au/TiO_2_ and transalkylation of the propyl side group to benzene with a zeolite catalyst. The limited phenol yield is owing to the low efficiency of the demethoxylation step, and the use of gold also raises concerns about the scale‐up of this process.

Earth‐abundant metals (e.g., Ni, Mo, Fe), which can replace noble metals, and their corresponding oxide, carbide, sulfide, and phosphide compounds have been reported as potential candidates for the deoxygenation of biomass‐derived molecules.^8^ Among them, transition‐metal phosphides show promising properties in the selective removal of oxygen in lignin‐derived molecules without hydrogenation of the aromatic ring. For example, 80 % of a gaseous guaiacol feed could be converted by using a supported Ni_2_P catalyst, with benzene (60 %) and phenol (30 %) as main products.^9^ In this work, we explored the potential of metal phosphides in the bifunctional upgrading of 2‐methoxy‐4‐propylphenol to phenol as a model reaction. Our approach was to first optimize the transition‐metal phosphide towards optimum HDMeO of 2‐methoxyl‐4‐propylphenol to 4‐propylphenol. MoP/SiO_2_ was identified as the most promising metal phosphide because the product yield was 88 mol % without touching the aromatic ring. The optimization of this catalyst in the bifunctional conversion of 2‐methoxyl‐4‐propylphenol to phenol led to a phenol yield close to 90 mol % at 350 °C at a high weight hourly space velocity (WHSV) of 40 h^−1^. The optimized system and conditions were then applied to convert an LFP‐derived lignin oil from pinewood into phenol. This strategy opens a new avenue in the valorization of biomass towards bulk chemicals, contributing to a greener chemical industry.

We first optimized the HDMeO function by comparing FeP/SiO_2_, CoP/SiO_2_, Ni_2_P/SiO_2_, WP/SiO_2_, and MoP/SiO_2_ using 2‐methoxy‐4‐propylphenol as a model compound. The metal phosphides were prepared by a two‐step wetness impregnation method of appropriate metal salts and diammonium hydrogen phosphate, followed by reduction at 700 °C aiming at the conversion of the metal oxide precursor to the corresponding phosphide (see the Supporting Information, Section 2.1). Silica is a preferred support for metal phosphides.^10^ Elemental analysis confirmed proper metal and phosphorus loadings and metal‐to‐phosphorus ratios (Table S1 in the Supporting Information). The formation of metal phosphides was evidenced by XRD (Figure S1 in the Supporting Information) and X‐ray photoelectron spectroscopy (XPS; Figure S2 in the Supporting Information).

The reduced catalysts were used to demethoxylate 2‐methoxy‐4‐propylphenol at 350 °C under 90 bar H_2_ pressure with benzene as the solvent (Figure 1).^7^, ^11^ The desired reaction is the removal of the methoxy group from the model reactant, which yields 4‐propylphenol and methanol. Removal of the phenolic hydroxyl group is more difficult and would lead to the formation of propylbenzene, and aromatic ring hydrogenation is another possible side reaction yielding propylcyclohexane. The reactant as well as the aromatic products can also be ring‐alkylated by methanol. The lowest product yield was obtained with the incompletely reduced Fe‐based catalyst. CoP/SiO_2_ converted 84 % of the reactant in the first hour with a 45 % yield of 4‐propylphenol, 18 % methylated 4‐propylphenols, and 6 % *n*‐propylbenzene. Although the product distribution was promising and aromaticity was retained, this catalyst suffered from severe deactivation. A higher and more stable conversion was attained with Ni_2_P/SiO_2_ as the catalyst. However, a drawback of this catalyst was that hydroxyl group removal and aromatic ring hydrogenation proceeded at substantial rates, leading to the formation of *n*‐propylcyclohexane. The higher aromatic ring hydrogenation rate also led to hydrogenation of the solvent, which is highly undesired (Figure S3 in the Supporting Information). WP/SiO_2_ showed a reasonable 4‐propylphenol yield of 40 mol % with a relatively small amount of methylated 4‐propylphenol byproducts. However, the conversion rate was low with this catalyst. The best performance was obtained with the MoP/SiO_2_ catalyst, enabling a near complete conversion of 2‐methoxy‐4‐propylphenol with an 88 mol % yield of 4‐propylphenol. Remarkably, this catalyst can remove the methoxy group without touching the phenolic hydroxyl group or the aromatic ring. The major byproducts were methylated 4‐propylphenols (10 mol %). Besides the nearly quantitative yield of alkylphenols, the catalyst also exhibited good stability during 6 h of reaction. Whereas the desirable selectivity can be correlated to the intrinsic catalytic properties of MoP, the high activity can be attributed to the significantly higher dispersion of the MoP phase on silica compared with the other catalysts, as judged from CO uptake measurements (Table S1 in the Supporting Information). In contrast to the other metal phosphides, TEM images show a high MoP dispersion (Figure S4 in the Supporting Information). The high dispersion of MoP can be related to the low mobility of (partially) reduced Mo phases (Table S2 in the Supporting Information).^12^, ^13^ Because earlier work indicated that MoO_3_ is also a potential catalyst for the deoxygenation of lignin monomers,^14^ we compared MoP/SiO_2_ to MoO_3_/SiO_2_ in our model HDMeO reaction. Although MoO_3_/SiO_2_ can also convert 2‐methoxy‐4‐propylphenol to the desired reaction product, MoP/SiO_2_ is much more active and produces fewer byproducts. These results highlight the unique ability of MoP/SiO_2_ for the selective removal of the methoxy group of 2‐methoxy‐4‐propylphenol by HDMeO.

**Figure 1:**
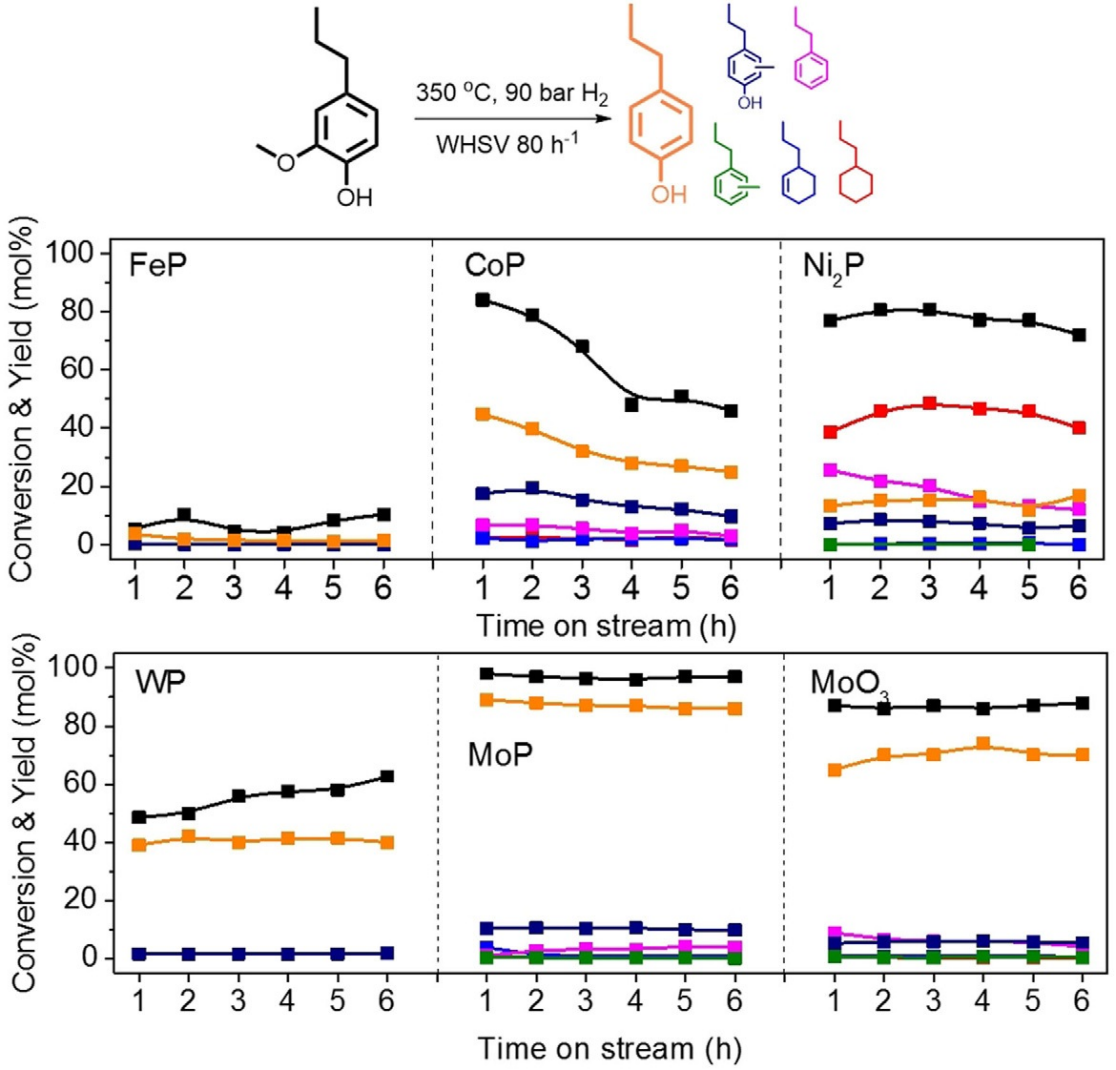
Conversion for 2‐methoxyl‐4‐propylphenol demethoxylation and product distribution over different metal‐phosphide catalysts: FeP/SiO_2_, CoP/SiO_2_, Ni_2_P/SiO_2_, WP/SiO_2_, MoP/SiO_2_, and MoO_3_/SiO_2_. Pretreatment: catalyst (100 mg) was reduced in 100 mL min^−1^ H_2_ at 450 °C for 1 h. Reaction conditions: 2‐methoxyl‐4‐propylphenol (5 mol %) in benzene, 350 °C, 90 bar, 30 mL min^−1^ H_2_, WHSV=80 h^−1^.

Obtaining phenol from the demethoxylated intermediate requires the removal of the alkyl group, for which we explored transalkylation to another aromatic molecule. Zeolites are shape‐selective catalysts for various kinds of alkylation/dealkylations reactions.^15^ These catalysts can also be involved in transalkylation, allowing a shift of alkyl groups from one aromatic molecule to another.^16^ We exploited this latter property by using benzene as a solvent, which is present in excess compared with the lignin monomer. In addition to the formation of valuable phenol, this approach also increases the value of the benzene stream because alkylated benzenes are more valuable than benzene in the chemical industry. We selected acidic HZSM‐5 with Si/Al=15 as a transalkylation catalyst. In a typical reaction, a mixture of passivated MoP/SiO_2_ (100 mg, particle size 75–200 μm) and HZSM‐5 (100 mg, particle size 300–500 μm) was placed in a stainless‐steel fixed‐bed reactor (Figure 2 a). Figure 2 b (left) shows the results of catalytic tests using a feed of 2‐methoxy‐4‐propylphenol and benzene (1:20 molar ratio) at 350 °C and 90 bar H_2_ at WHSV=40 h^−1^. The combined demethoxylation by MoP/SiO_2_ and transalkylation by zeolite resulted in a promising phenol yield of 83 % after 1 h. The propyl chain was mainly transferred to benzene, yielding *n*‐propylbenzene and cumene. These products have particular value in the current context because they can be converted to phenol by oxidation.^17^ Toluene and xylenes were other reaction products, which were most likely obtained by alkylation of benzene with methanol derived from the removal of the methoxy group as well as isomerization reactions of cumene and *n*‐propylbenzene. Furthermore, the observation of diphenylmethane (3 mol %) points to condensation reactions, which could explain the slow accumulation of heavy deposits (Figures S5 and S6 in the Supporting Information). Propane (1 mol %) was found to be dissolved in benzene, whereas a small amount of methane was formed in the gas phase owing to the propyl guaiacol demethylation catalyzed by HZSM‐5.^7^ Although the initial phenol yield was high during the first hour, the catalyst deactivated, as evident from the slowly decreasing phenol yield. This was clearly owing to a loss of the transalkylation activity because the 4‐propylphenol yield increased with time on stream (Figure 2 b left). A similar trend was observed among benzene‐derived products, for which the propylbenzene yield became lower with time on stream (Figure S7 in the Supporting Information).

**Figure 2:**
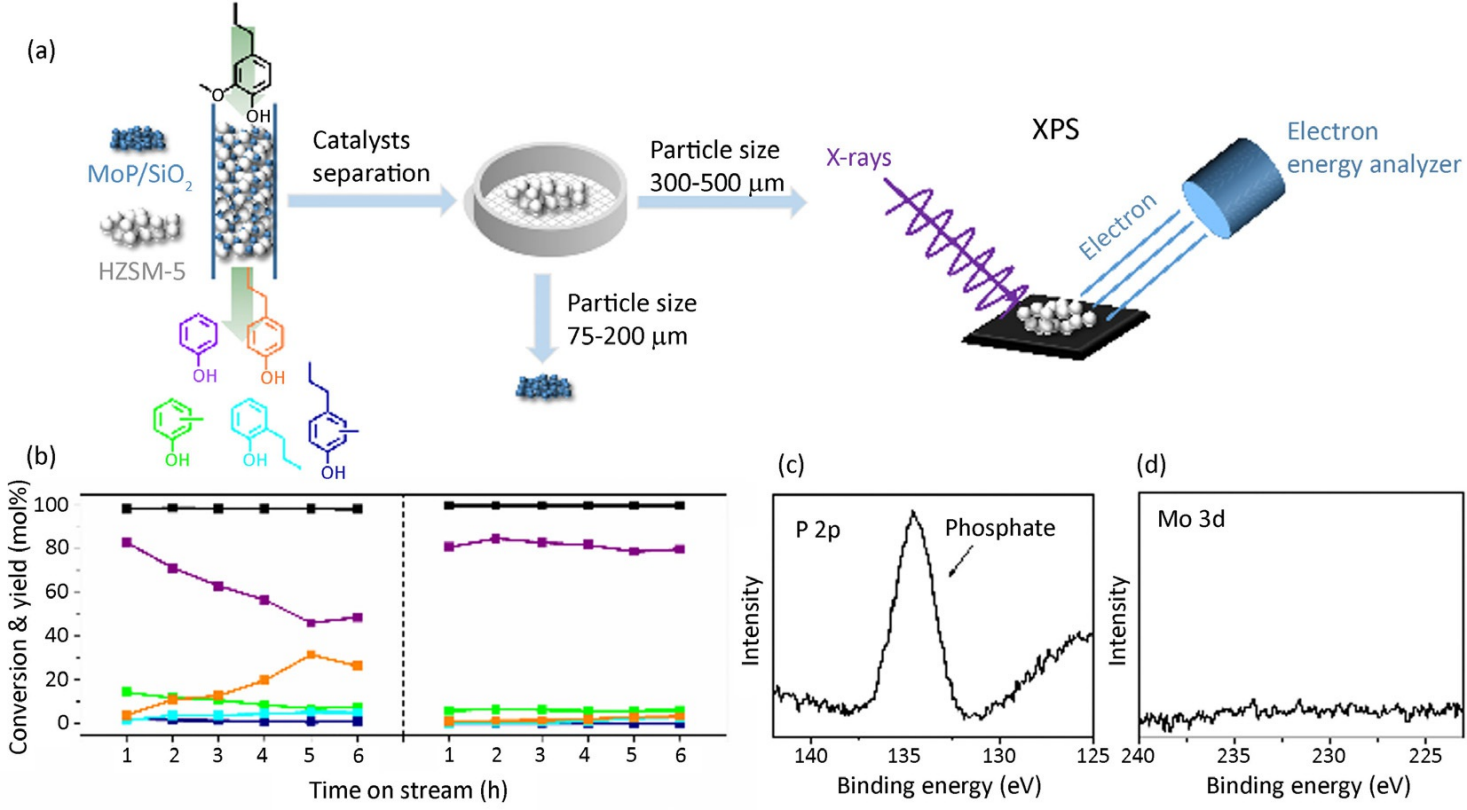
(a) One‐pot conversion of 2‐methoxy‐4‐propylphenol to phenol in a fixed‐bed flow reactor, separation of the two catalytic components of the used catalysts, and XPS analysis of used HZSM‐5; (b) conversion and product yield using HZSM‐5 with passivated MoP/SiO_2_ (left) and non‐passivated MoP/SiO_2_ (right). Pretreatment: reduction of MoP/SiO_2_ (100 mg) and HZSM‐5 (100 mg) at 450 °C for 1 h. Reaction conditions: 2‐methoxyl‐4‐propylphenol (5 mol %) in benzene, 350 °C, 90 bar, 30 mL min^−1^ H_2_, WHSV=40 h^−1^. (c) P 2p and (d) Mo 3d XPS spectra of used HZSM‐5 catalyst.

To investigate the origin of this undesired activity loss, the used HZSM‐5 zeolite was separated from the catalyst mixture by sieving (different particle sizes were used for the two catalyst components) and analysis by XPS (Figure 2 a). XPS shows that the used zeolite contains phosphate species as evident from the P 2p signal at 134.9 eV (Figure 2 c). Importantly, the zeolite component does not contain molybdenum nor other (reduced) phosphide species (Figure 2 d). Thus, we can firmly conclude that unreduced phosphate left over from the preparation migrated from the MoP/SiO_2_ component to HZSM‐5 during the ongoing reaction. Although phosphate is regarded as a promoter for the hydrothermal stability of HZSM‐5, it strongly interacts with Brønsted acid sites of zeolites, lowering the total acidity and thus the activity.^18^ This deactivation mechanism was confirmed by the poor performance (lower conversion and phenol yield) of a phosphate‐modified HZSM‐5 in comparison to a P‐free HZSM‐5 zeolite in 4‐propylphenol transalkylation (Figure S8 in the Supporting Information). Thus, we can conclude that the zeolite acid sites are deactivated by phosphate species migrating from the MoP/SiO_2_ to HZSM‐5. We also considered that these phosphate species can originate from the passivation procedure of the MoP phase during the reduction step and possibly the further exposure in ambient air when loading the reactor. To verify this, we developed a protocol in which the reduced MoP/SiO_2_ was directly mixed with the zeolite catalyst and loaded into the reactor in a nitrogen‐flushed glovebox. Figure 2 b (right) shows that this approach yielded a more stable catalyst system for phenol production. A high and stable phenol yield of approximately 80 % was obtained with cresol as the main byproduct. The alkylation of benzene in this modified approach was confirmed, and the yield of alkylated products with time on stream was also stable (Figure S7 in the Supporting Information). Thus, preventing MoP oxidation, which would otherwise generate phosphate species, is a key factor in obtaining a stable catalyst for a high phenol yield.

Recognizing the importance of avoiding phosphate, we optimized the preparation of MoP by varying the reduction temperature required to obtain the phosphide from Mo oxide and phosphate precursors in the 600–900 °C range (Figures S9–S11 in the Supporting Information). The catalytic performance of these differently reduced MoP/SiO_2_ catalysts was evaluated in combination with HZSM‐5 for the conversion of 2‐methoxy‐4‐propylphenol to phenol. The conversion in these tests was complete, and the phenol yield was very high with typical cresol yields of approximately 10 mol % (Figure S12 in the Supporting Information). There was no observable deactivation during these measurements, as also confirmed by the near constant yield of *n*‐propylbenzene and cumene (Figure S13 in the Supporting Information). Despite the minor effect on the conversion, Figure 3 shows that a higher reduction temperature results in a higher phenol yield. The higher degree of MoP formation and the slightly lower amount of residual phosphate can contribute to these improvements. XPS analysis of used MoP/SiO_2_ catalysts shows that the Mo oxidation state did not change during the reaction (Figure S14 in the Supporting Information). Nevertheless, we still observed a small loss of phosphate species from the decrease of the corresponding XPS P 2p feature. The phosphorus content on used zeolite was very high (0.7 wt %) in the experiment in which MoP/SiO_2_ was reduced at 600 °C and lowest in the experiment in which reduction was performed at 900 °C (0.4 wt %) (Figures S15 and S16 in the Supporting Information).

**Figure 3:**
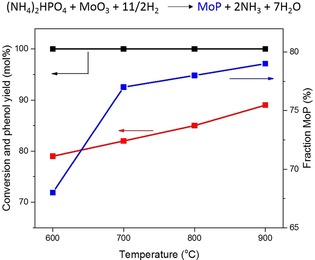
Influence of reduction temperature on phosphide formation (blue), 2‐methoxy‐4‐propylphenol conversion (black), and phenol yield (red, average yield during 6 h reaction).

The above results show the promise of using a combination of MoP/SiO_2_ and HZSM‐5 zeolite in converting lignin‐derived guaiacol‐type compounds in high yield to phenol. We also evaluated the stability of this catalyst combination in a 48 h reaction (Table S3 in the Supporting Information). The phenol yield was found to slowly decrease with time owing to coke formation. After catalyst regeneration at 700 °C for 2 h in a hydrogen atmosphere, the phenol yield was 85 % again. The utility of this catalyst combination in converting a syringol‐type 2,6‐dimethoxy‐4‐propylphenol in separate demethoxylation and transalkylation reactions showed that the phenol yield in this case was much lower at 31 mol % with a non‐closed mass balance, likely owing to oligomerization of intermediates (Tables S4 and S5 in the Supporting Information).

Although further investigations are needed to more efficiently convert syringol‐type intermediates coming from hardwood biomass, we decided to evaluate the potential of our approach on a real lignin oil derived from pinewood by LFP (Figure 4). We chose pinewood because this kind of softwood is rich in guaiacol‐type building blocks. For the LFP, pinewood sawdust was subjected to a reaction at 230 °C and 30 bar H_2_ using a Pt/C catalyst in a methanol/water mixture (molar methanol/water ratio=1:2).^19^ All β‐O‐4 lignin interlinkages in the original biomass were cleaved, as confirmed by heteronuclear single quantum coherence (HSQC) NMR spectroscopy (Figure S19 in the Supporting Information). Gel permeation chromatography evidenced the formation of lignin monomers with only a small amount of relatively low‐molecular‐weight lignin fragments dissolved in benzene (Figure S20 in the Supporting Information). The total monomer yield was 15 wt %, of which 78 % was 2‐methoxy‐4‐propylphenol. Other lignin monomers were guaiacol, methylguaiacol, and ethylguaiacol. The lignin monomers in this oil were then further converted by our combination of MoP/SiO_2_ and HZSM‐5 catalysts (Figure S21 in the Supporting Information). Not only 4‐propylguaiacol but also ethylguaiacol and methylguaiacol were converted to phenol, showing that our approach is able to broadly convert substituted guaiacols. Importantly, full conversion of these compounds in the lignin oil and stable phenol yield support our conclusion that the dual catalyst is stable for at least 6 h. Based on the initial lignin content in pinewood, we determined a phenol yield of 9.6 mol %. In summary, we developed a novel catalytic approach for valorization of lignin monomers obtainable from different types of wood by LFP. In a relevant example, lignin in pinewood was converted to phenol using a combination of reductive depolymerization of in planta lignin followed by combined hydrodemethoxylation and transalkylation. The upgrading of the carbohydrate pulp to value‐added products through different strategies has already been explored before.^20^ The novelty of our approach is the use of relatively cheap MoP/SiO_2_ to catalyze hydrodemethoxylation. We demonstrated that the formation of MoP species is not only important for a high demethoxylation activity but also essential to limit the amount of remaining phosphate precursor to stabilize the performance. A too‐high residual phosphate yield leads to slow deactivation of the zeolite component owing to migration to Brønsted acid sites. Conceptually, an advantage of the described approach is the benefit of integrating the described novel biobased process in existing chemical processes for phenol production.

**Figure 4:**
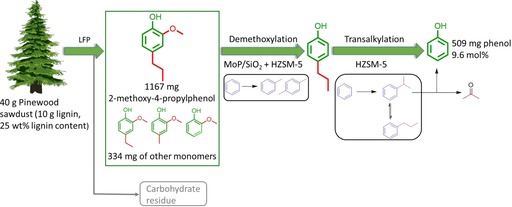
Schematic representation of the three‐step approach developed in this work to obtain phenol from woody biomass.

## Conflict of interest

*The authors declare no conflict of interest*.

## Supporting information

As a service to our authors and readers, this journal provides supporting information supplied by the authors. Such materials are peer reviewed and may be re‐organized for online delivery, but are not copy‐edited or typeset. Technical support issues arising from supporting information (other than missing files) should be addressed to the authors.

Supplementary
