# Supplementary material for: Efficient Conversion of Pine Wood Lignin to Phenol
Source: ChemSusChem. 2020 Mar 10;13(7):1705–9. doi: 10.1002/cssc.202000485 (PMC7187360; doi:10.1002/cssc.202000485)
Supplement: Supplementary file 1 — Supplementary [file CSSC-13-1705-s001.pdf]

CHEMISTRY & SUSTAINABILITY
CHEMSUSCHEM
ENERGY & MATERIALS

# Supporting Information

## Efficient Conversion of Pine Wood Lignin to Phenol

Xianhong Ouyang,[a] Xiaoming Huang,*[a, c] Michael D. Boot,[b] and Emiel J. M. Hensen*[a]

cssc_202000485_sm_miscellaneous_information.pdf

Part A: Experimental procedures

Part B: Figures

Part C: Tables

Part D: References

# A. Experimental procedures

## 1. Chemicals and materials

The metal salt precursors $Ni(NO)_3·6H_2O$, $Fe(NO_3)_2·9H_2O$, $(NH_4)_6Mo_7O_{24}·4H_2O$, $Co(NO_3)_2·6H_2O$ (99.999%) were purchased from Sigma-Aldrich. The phosphorus precursor $(NH_4)_2HPO_4$ was purchased from Merck. Silica, 5 wt% Pd/C and 5 wt% Pt/C were purchased from Sigma-Aldrich. Zeolite $NH_4$ZSM-5 (Si/Al 15) was obtained from Albemarle. Model compounds 4-propylphenol (⩾97%), 2-methoxy-4-propylphenol (⩾99%) and 4-allyl-2,6-dimethoxyphenol (⩾95%) were bought from Sigma-Aldrich. Benzene (99.7%) was bought from VWR. Tetrahydrofuran (THF), and $d_6$-DMSO (dimethylsulfoxide) were purchased from Merck. n-Dodecane was purchased from Alfa Aesar.

## 2. Catalyst preparation

### 2.1 Transition metal phosphides

Transition metal phosphide catalysts were prepared by a two-step incipient impregnation method. First, the silica supports were impregnated with an aqueous solution of $Ni(NO)_3·6H_2O$, $Fe(NO_3)_2·9H_2O$, $(NH_4)_6Mo_7O_{24}·4H_2O$, $Co(NO_3)_2·6H_2O$ and $(NH_4)_6W_{12}O_{39}·H_2O$, respectively. The metal loading was 1.6 mmol/g $SiO_2$ support. The impregnated catalysts were dried in an oven at 105 °C overnight and calcined at 550 °C for 5 h. The obtained metal oxide catalysts were impregnated with an aqueous solution of $(NH_4)_2HPO_4$. The targeted phosphorus/metal ratio was 1/1 for $WP/SiO_2$, $CoP/SiO_2$, $MoP/SiO_2$ and 2/1 for $Ni_2P/SiO_2$ and $Fe_2P/SiO_2$. After drying in an oven at 105 °C overnight, these catalysts were reduced in 100 mL/min $H_2$ flow at 700 °C for 3 h (heating rate 3 °C/min). After reduction, the silica-supported metal phosphide catalysts were passivated in 0.5 % vol $O_2$ in Ar for 2 h. The synthesis of non-passivated $MoP/SiO_2$ catalyst is similar to the procedure described in section 2.1. After reduction at different temperatures (600, 700, 800, 900 °C) for 3 h (heating rate 3 °C/min), the obtained $MoP/SiO_2$ samples were transferred into a glovebox without passivation. A $MoO_3/SiO_2$ catalyst was synthesized by an incipient impregnation method using $(NH_4)_6Mo_7O_{24}·4H_2O$ as the precursor. After drying and calcination, the catalyst was reduced in 100 mL/min $H_2$ flow at 700 °C for 3 h (heating rate 3 °C/min). After reduction, the $MoO_3/SiO_2$ catalysts was passivated in 0.5 % vol $O_2$ in Ar for 2 h.

### 2.2 Zeolite

$NH_4$ZSM-5 zeolite was calcined at 550 °C for 6 h (heating rate of 1 °C/min) to obtain the proton form (HZSM-5).

## 3. Catalytic Activity Test

### 3.1 HDMeO 2-methoxy-4-propylphenol

A home-built down-flow fixed-bed reactor was used for the HDMeO of the lignin monomer model compound 2-methoxy-4-propylphenol. Typically, 100 mg of passivated metal phosphide catalyst (sieve fraction 75-200 μm) was loaded in a tubular reactor. The catalyst was pretreated in a $H_2$ flow (100 ml/min) at 450 °C for 1 h (heating rate 3 °C /min). After pretreatment, the temperature was decreased to 350 °C and the $H_2$ flow rate was set to 30 mL/min. The pressure was raised to 90 bar by a back-pressure regulator. After reaching the reaction pressure and temperature, a feed of 5 mol% 2-methoxy-4-propylphenol in

benzene was fed to the reactor through an HPLC pump at a flow rate of 0.15 mL/min. The feeding line between the HPLC pump and reactor was heated to 200 °C. The liquid products were collected after the reactor every 1 hour in a cold trap. 1 mL of liquid was taken out of the product mixture. After adding 10 μL n-dodecane as an external standard, the product yield is analyzed by a Shimadzu 2010 gas chromatograph with mass spectrometry and flame ionization detection equipped with a TRX- 1701 column.

3.2 HDMeO and transalkylation 2-methoxy-4-propylphenol

The reactor described above was used for obtaining phenol from 2-methoxy-4-propylphenol. Typically, 100 mg of passivated MoP/$SiO_2$ catalyst (75-200 μm) and 100 mg of HZSM-5 (Si/Al 15, 300-500 μm) were mixed and loaded in a tubular reactor equipped with two valves at the top and bottom. The reaction conditions and procedures were the same as described in section 3.1. After reaction, the tubular reactor was sealed by closing the valves. The used catalysts were transferred to a glovebox and separated via sieving without contacting air. The used catalysts were further characterized.

For evaluating the performance of non-passivated MoP/$SiO_2$ catalyst, 100 mg MoP/$SiO_2$ catalyst (75-200 μm) and 100 mg HZSM-5 (Si/Al 15, 300-500 μm) were mixed and loaded in a tubular reactor in the glovebox. After closing the valves, the reactor was transferred and connected to the setup. The reaction conditions and procedures were the same as described in section 3.1. After reaction, the tubular reactor was sealed by closing the valves. The used catalysts were transferred to a glovebox and separated via sieving without contacting air followed by further characterization.

The reactant conversion and product yield was calculated as follows:

$$Conversion = \left(1 - \frac{moles\ of\ 2 - methoxy - 4 - propyl\ phenol\ after\ reaction}{initial\ moles\ of\ 2 - methoxy - 4 - propyl\ phenol}\right) \times 100\%$$

$$Yield = \frac{moles\ of\ product}{initial\ molar\ of\ 2 - methoxy - 4 - propyl\ phenol} \times 100\%$$

The solvent-derived product yield was calculated as follows:

$$Solvent\ derived\ products\ yield = \frac{moles\ of\ solvent\ derived\ product}{initial\ moles\ of\ solvent} \times 100\%$$

3.3 Conversion 4-propyl-2,6-dimethoxyphenol

3.3.1 Synthesis 4-propyl-2,6-dimethoxyphenol

Commercially available -allyl-2,6-dimethoxyphenol was hydrogenated to obtain the 4-propyl-2,6-dimethoxyphenol. In a typical reaction, 3.0 g of 4-allyl-2,6-dimethoxyphenol, 100 mg of 5wt% Pd/C and 40 mL of methanol were loaded in a Parr autoclave. The autoclave was sealed and flushed with nitrogen. After leak testing, the autoclave was pressurized to 30 bar with $H_2$ at room temperature. The mixture was stirred at 500 rpm and heated to 40 °C. The reaction time was 4 hours. After reaction, the autoclave was cooled in ice water. After releasing pressure, the reaction mixture and Pd/C catalyst were separated by centrifuge. The reaction mixture was analyzed by GC and GC-MS. The syringol-type monomer 4-propyl-2,6-dimethoxyphenol was obtained by methanol evaporation. GC-MS data are showed in figure S17.

3.3.2 HDMeO and transalkylation 4-propyl-2,6-dimethoxyphenol

Demethoxylation of 4-propyl-2,6-dimethoxyphenol was carried out in a Parr autoclave in the first step. In a typical reaction, 1 g (5.1 mmol) of 4-propyl-2,6-dimethoxyphenol, 100 mg of $MoP/SiO_2$ (synthesized at 700 °C without passivation and kept in glovebox) and 40 mL of benzene were loaded in a Parr autoclave. The autoclave was sealed and flushed with nitrogen. After a leak test, the autoclave was pressurized to 50 bar with $H_2$ at room temperature. The mixture was stirred at 500 rpm and heated to 350 °C for 2 hours. After reaction, the autoclave was cooled to room temperature. After releasing the pressure, the reaction mixture and $MoP/SiO_2$ were separated by filtration. To determine the conversion and product yield, 1 µL of n-dodecane was added as an external standard to 1 mL of the reaction mixture, which was then analyzed by GC and GC-MS.

Transalkylation was carried out in the same Parr autoclave. The reaction mixture after demethoxylation and 100 mg of HZSM-5 (Si/Al 15) were loaded in a Parr autoclave. The autoclave was sealed and flushed with nitrogen. After a leak test, the autoclave was pressurized to 50 bar with $H_2$ at room temperature. The mixture was stirred at 500 rpm and heated to 350 °C for 2 hours. After reaction, the autoclave was cooled to room temperature. After releasing pressure, the reaction mixture and zeolite were separated by filtration. To determine the conversion and product yield, 1 µL of n-dodecane was added as an external standard to 1 mL of reaction mixture, which was then analyzed by GC and GC-MS. Product yield was determined as follow:

$$Conversion\ (mol\%) = \left(1 - \frac{moles\ of\ reactant\ after\ reaction}{moles\ of\ reactant\ before\ reaction}\right) \times 100\%$$

$$Yield\ (mol\%) = \frac{moles\ of\ product\ after\ reactoin}{moles\ of\ initial\ reactant} \times 100\%$$

## 4. Catalyst Characterization

Powder X-ray diffraction (XRD) was measured on a Bruker Endeavor D2 with Cu Kα radiation (40 kV and 30 mA). The XRD pattern was recorded with 0.02° steps over the 10° - 80° angular range with 0.4 s per step.

Transmission electron microscopy (TEM) was used to determine the particle size and particle size distribution. A catalyst sample was ground and suspended in the ethanol for the TEM analysis. Images were taken using an FEI Tecnai 20 at 200 kV. Particle size distribution analysis was carried out in the ImageJ software.

Textural properties of the silica-supported metal phosphide catalysts were determined by analyzing nitrogen physisorption isotherms recorded at -196 °C on a Micromeritics ASAP3020 Tristar system. Typically, 100 mg of sample was loaded in a quartz tube which was pretreated at 120 °C overnight under dynamic vacuum to remove the water prior to analysis. The Brunauer-Emmet-Teller (BET) method was used to calculate the surface area.

The metal and phosphorus loading was determined by inductively coupled plasma atomic emission spectrometry (ICP-AES) analysis performed on a SpectroBlue apparatus. Before analysis, the samples (20 mg) were dissolved in an acid mixture (1.5 mL) of $HF(40\%)/HNO_3(65\%)/H_2O$ in a 1/1/1 volumetric ratio. The solution was transferred to a volumetric flask of 50 mL. After diluting ten times by pure water, the samples were analyzed by ICP-AES.

CO uptake measurements were used to determine the dispersion of metal atoms. Typically, 0.3 g silica supported metal phosphide catalyst was loaded into a quartz reactor. The samples were reduced in flowing $H_2$ at 450 °C (heating rate 10 °C/min) and evacuated at 450 °C for 1 hour to remove chemisorbed hydrogen, and then cooled to 40 °C under dynamic vacuum. Chemisorption analysis was carried out at 40 °C.

X-ray photoelectron spectroscopy (XPS) measurements were performed on a Thermo Scientific K-Alpha XPS equipped with a monochromatic Al Kα X-ray. For the measurement of passivated metal phosphide catalysts, all the samples were reduced in an $H_2$ flow (100 mL/min) at 450 °C for 1 hour (heating rate 3 °C /min). After reduction, the samples were transferred to glovebox and ground without exposure to air. The ground samples were loaded in a vacuum transfer cell for the XPS analysis. For the analysis of non-passivated and used catalysts, the samples were directly ground and loaded in the transfer cell in the glovebox for the XPS analysis. Fitting of the XPS spectroscopy was done with CasaXPS software. The carbon 1s line at 284.5 eV was used for energy calibration.

**5. Biophenol production from pinewood sawdust**

5.1 Pinewood sawdust pretreatment

Pinewood sawdust was pretreated with water (24 h) and ethanol (24 h) in a Soxhlet reactor to remove the extractives. After pretreatment, the sawdust was dried at 105 °C overnight.

5.2 Klason lignin content determination

The Klason lignin content (25 wt%) in pinewood was determined by a two-step $H_2SO_4$ hydrolysis procedure.[1] Typically, 300 mg of the pine sawdust and 3 mL of 72% sulfuric acid were loaded in a pressure tube. Then, the tube was placed in a water bath set at 30 °C and incubated for 60 minutes. During the first hydrolysis, the sample was stirred by a rod every 5 min without removing the sample from water bath. After the first step, the acid concentration was diluted to 4% by adding 84 mL of purified water into the pressure tube. After screwing the Teflon cap, the tube was placed in an oil bath set at 121 °C for 1 hour to complete the second hydrolysis.

After the two-step hydrolysis, the solid was collected by filtration and dried at 105 °C in an oven for 4 hours. The weight of dried solid was recorded. The dried solid was then placed in a calcination oven set at 575 °C to determine the ash content.

The Klason lignin content in birch wood is calculated as follow.

$$\text{Klason lignin content (\%)} = \frac{\text{weight of dried solid after hydrolysis} - \text{weight of ash}}{\text{weight of birch wood sample}} \times 100\%$$

5.3 Pinewood sawdust lignin depolymerization

40 gram of pretreated pine sawdust, 800 mL of solvent (methanol 423 mL, water 377 mL), 2 g of 5 wt% of Pt/C catalyst were loaded in a 4 L autoclave. The autoclave was sealed and flushed three times with nitrogen to remove air. After a leak test, the autoclave is pressurized to 30 bar with $H_2$. The reaction mixture was heated to 230 °C (heating rate ~2 °C/min). After 3 hours reaction, the autoclave was cooled to room temperature. The lignin oil and solid residue were separated by filtration. The methanol/water solvent was removed by a rotary evaporator. 423 mL of ethyl acetate and 377 mL of water were added to the lignin oil. Lignin products were dissolved in organic phase while sugar-derived products were kept in the water phase. 40 mL of ethyl acetate phase was taken for further GC/GC-MS, GPC and 2D HSQC NMR

analysis. After this liquid-liquid extraction, the ethyl acetate was separated and then removed by rotary evaporation. The obtained lignin oil was further dissolved in benzene for biophenol production. Lignin monomer yield is calculated by GC/GCMS and monomer concentration is 0.5 mol% in benzene.

5.4 Biophenol production

$MoP/SiO_2$ catalyst was synthesized at 700 °C without passivation (details in suporting information 2.1). Typically, 100 mg of $MoP/SiO_2$ catalyst and 100 mg HZSM-5 (Si/Al 15) were mixed and loaded in a fixed-bed reactor in a glovebox. The reactor was sealed by two valves before it was taken out of the glovebox. The catalysts were pretreated in an $H_2$ flow (100 mL/min) at 450 °C for 1 h (heating rate 3 °C/min). After pretreatment, the temperature was decreased to 350 °C and the $H_2$ flow rate was set to 30 mL/min. The pressure was controlled at 90 bar by a back-pressure regulator. After reaching the reaction pressure and temperature, the lignin oil solution (0.5 mol% monomers in benzene) was fed into the reactor through an HPLC pump at a flow rate of 0.15 mL/min. The feeding line between the HPLC pump and reactor was heated to 200 °C. The liquid products were collected after the reactor every 1 hour in a cold trap. To analyze the conversion and yield, 1 mL of liquid was taken out of the product mixture. After adding 5 µl dodecane as an external standard, the products yield is analyzed by GC/GCMS.

$$Conversion = \left(1 - \frac{moles\ of\ monomers\ after\ reaction}{moles\ of\ monomers}\right) \times 100\%$$

$$Yield = \frac{moles\ of\ phenol}{moloes\ of\ monomers} \times 100\%$$

In the above definition, the amount of monomers is the amount of moles of phenolic monomers obtained in pinewood lignin depolymerization as described in supporting information 5.3.

To quantify the phenol yield (mol%) based on the initial lignin content, we used HSQC NMR to determine the S/G/H ratio in the lignin oil.[2] The monomer composition of the lignin oil was determined by integration of the $S_{2,6}$, $H_{2,6}$ and $G_2$ correlation signals (Figure S14). For pinewood lignin oil obtained in Pt/C catalyzed depolymerization, the S unit signals were not observed and the G/H ratio was 91/9. The aromatic building blocks in pinewood are mainly *p*-coumaryl, coniferyl alcohols with molecular weights of 150 and 180 g/mol, respectively. Therefore, we can estimate the average molecular weight of monomers in pinewood lignin oil is 177.3 g/mol. We used 40 g of pinewood with 25 wt% of lignin content, which represents 56.4 mmol of initial lignin monomers. The phenol yield (mol%) based on the initial lignin content is calculated as follow.

$$\text{Phenol yield} = \frac{moles\ of\ phenol}{moles\ of\ initial\ lignin\ monomers\ in\ pinewood} \times 100\%$$

## B. Figures

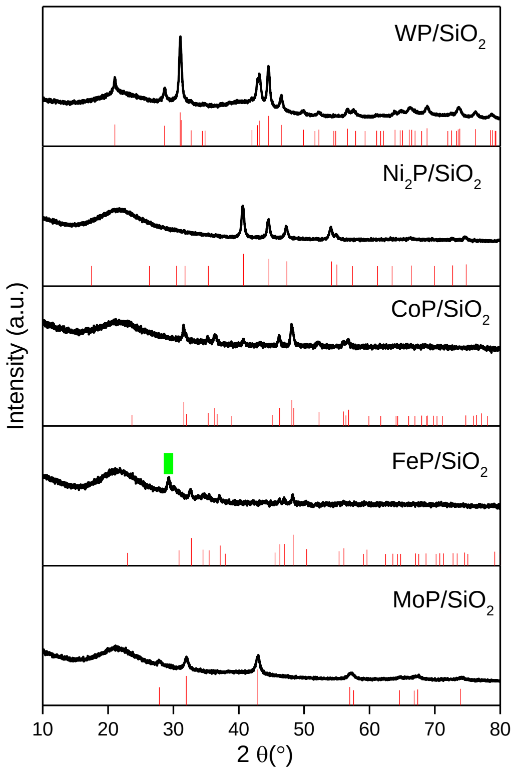


**Figure S1** XRD pattern of silica-supported transition metal phosphide catalysts. Red lines are the peaks corresponding the different metal phosphides from the powder diffraction file database. The peak at the green square position indicates the formation of $Fe_2P_2O_7$. XRD confirms the formation of CoP, $Ni_2P$, WP and MoP, whereas complete reduction to FeP was not achieved as can be judged from the presence of $Fe_2P_2O_7$ reflections in the XRD pattern.

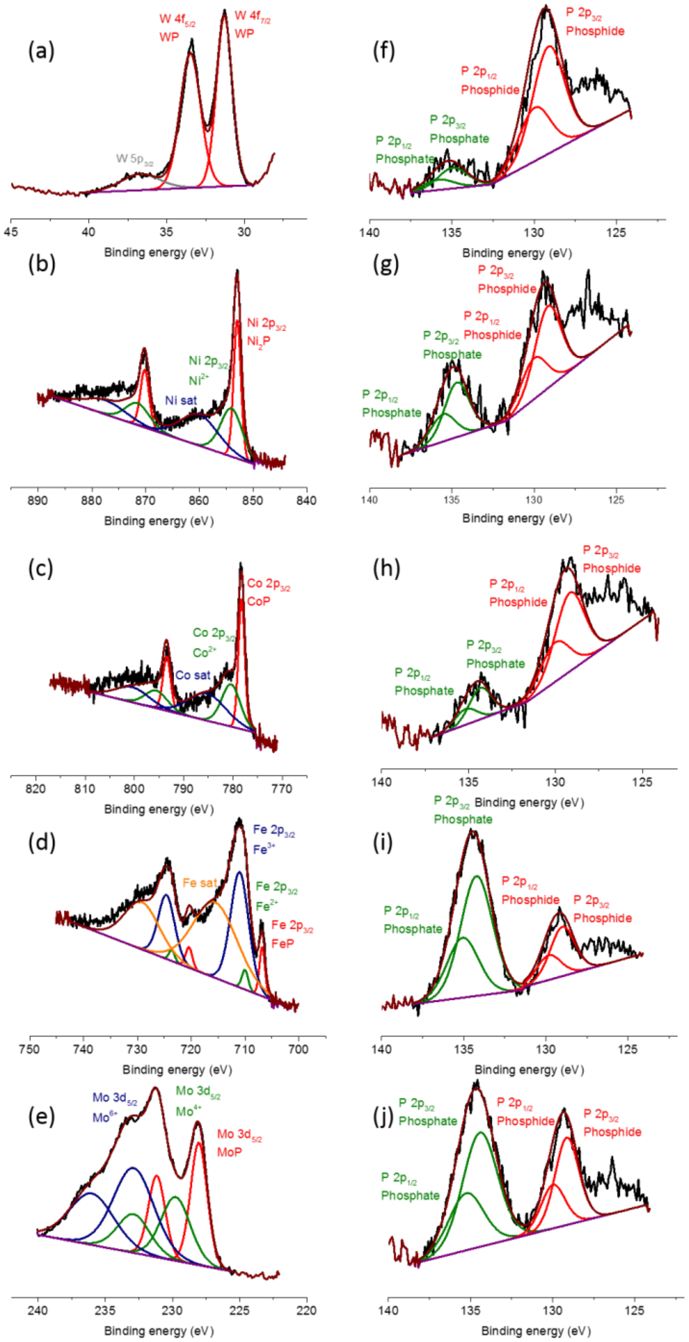


**Figure S2** XPS spectra of (a) W 4f, (b) Ni 2p, (c) Co 2p, (d) Fe 2p, (e) Mo 3d and their corresponding P 2p (f-j) in metal phosphide catalysts synthesized at 700 °C. The passivated metal phosphide catalysts were reduced in 100 mL/min $H_2$ at 450 °C for 1 hour and then analyzed by XPS. XPS spectra demonstrate the formation of metal and phosphorus species with an oxidation state close to zero upon high-temperature reduction. On the other hand, there are only very weak signals visible of reduced Fe and P (Fe $2p_{3/2}$ 707 eV, P $2p_{3/2}$ 129.0 eV).

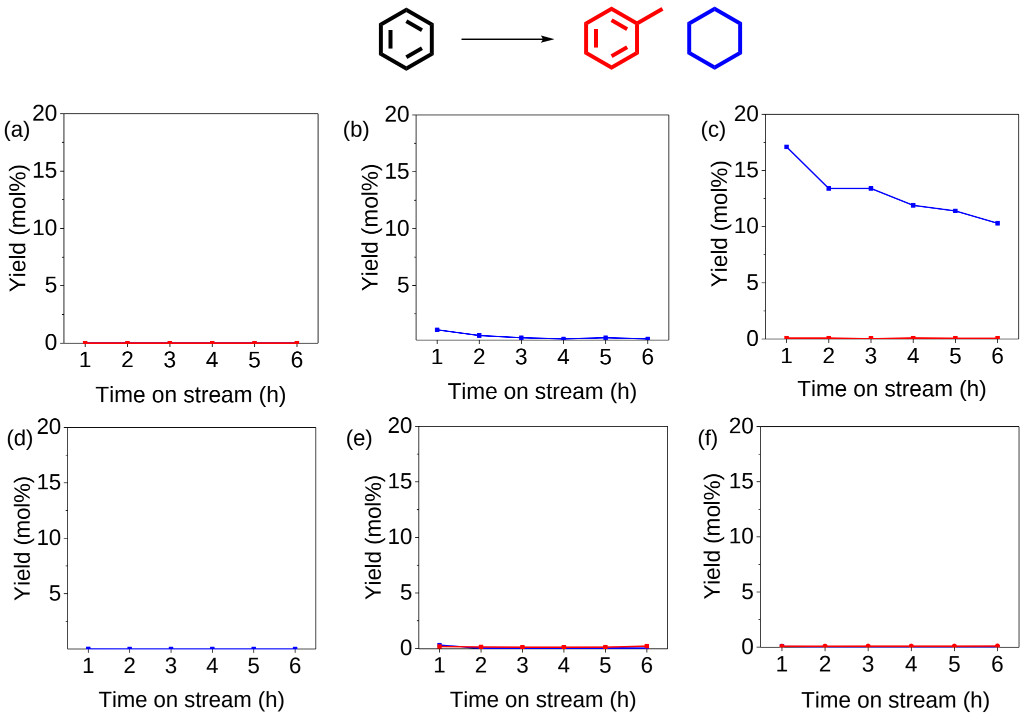


**Figure S3** Solvent-derived products in the HDMeO of 2-methoxyl-4-propylphenol over different metal phosphide catalysts: (a) FeP/$SiO_2$; (b) CoP/$SiO_2$; (c) $Ni_2P$/$SiO_2$; (d) WP/$SiO_2$; (e) MoP/$SiO_2$ and (f) $MoO_3$/$SiO_2$ catalyst. Pretreatment conditions: 100 mg of catalyst reduced in 100 mL/min $H_2$ at 450 °C for 1 hour. Reaction conditions: 5 mol% of 2-methoxyl-4-propylphenol in benzene, 350 °C, 90 bar, gas flow rate 30 mL/min $H_2$, weight hourly space velocity (WHSV) 80 $h^{-1}$.

**Figure S4** TEM images of silica-supported transition metal phosphide catalysts reduced at 700°C (a) $WP/SiO_2$, (b) $Ni_2P/SiO_2$, (c) $CoP/SiO_2$, (d) $FeP/SiO_2$, (e) $MoP/SiO_2$.

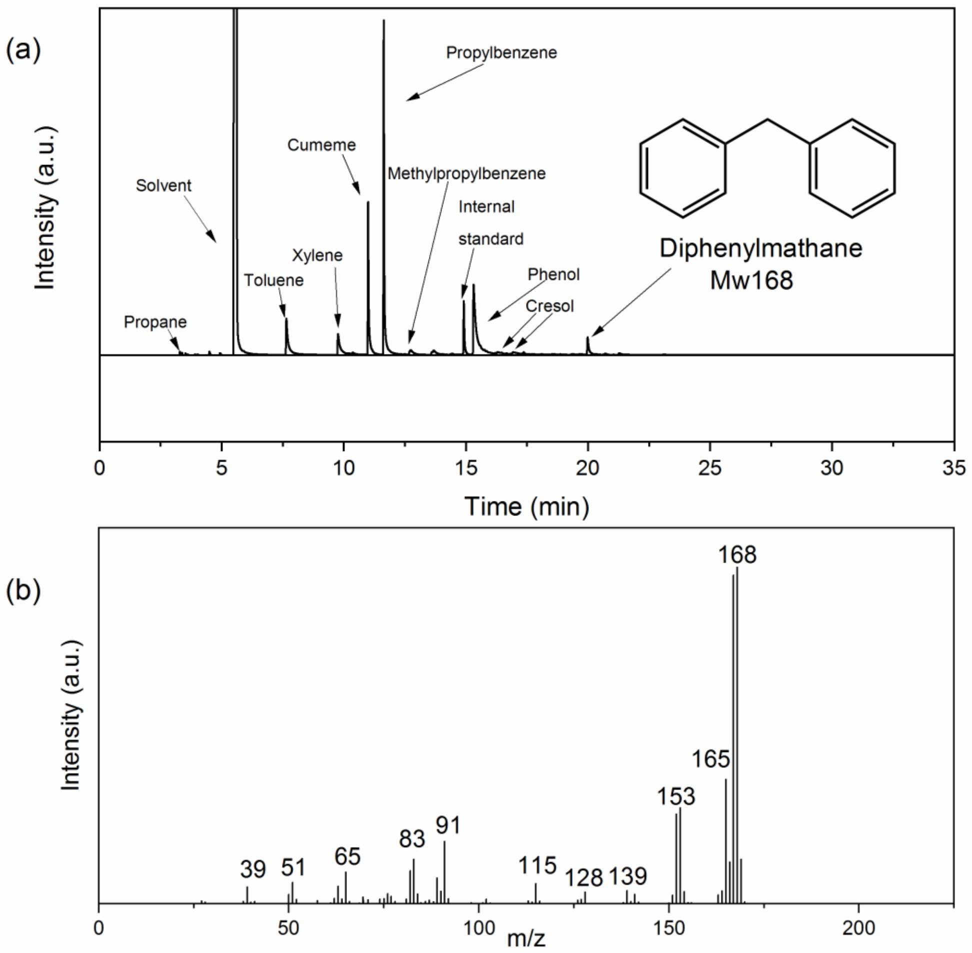


**Figure S5** Products of 4-propyl-2-methoxy phenol conversion using HZSM-5 and passivated MoP/SiO2. (a) GCMS analysis of the first-hour sample; (b) MS of diphenylmethane.

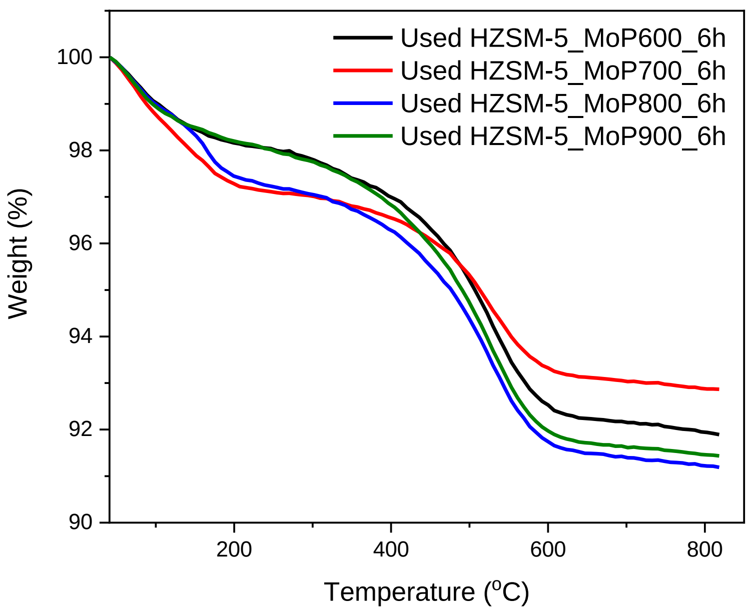


**Figure S6** TG analysis of used HZSM-5 catalysts. HZSM-5 was separated from the catalyst mixture by sieving after the reaction.

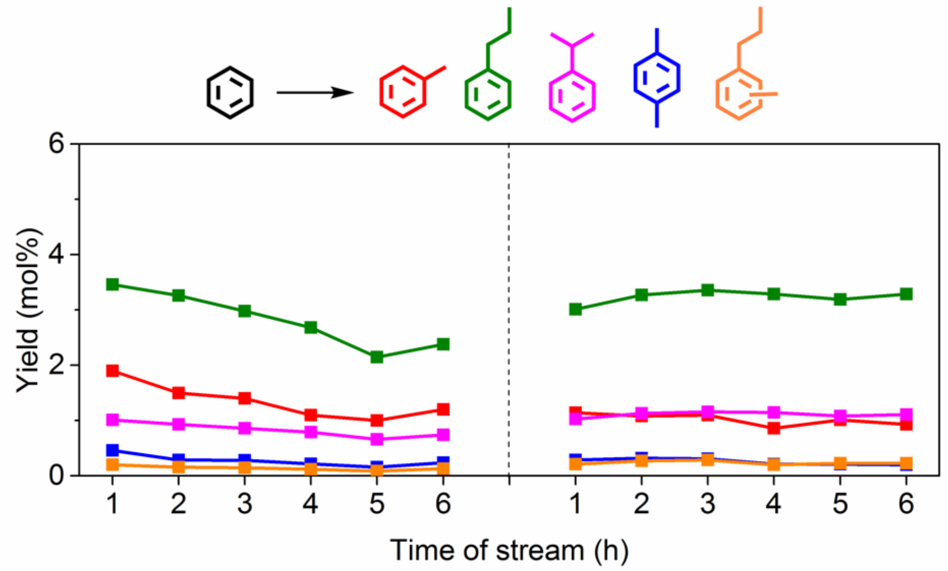


**Figure S7** Benzene-derived products in the transalkylation of 2-methoxy-4-propylphenol with benzene using HZSM-5 and (left) passivated MoP/$SiO_2$ and (right) non-passivated MoP/$SiO_2$. Pretreatment conditions: the mixture of MoP/$SiO_2$ and HZSM-5 is reduced in 100 mL/min $H_2$ at 450 °C for 1 hour. Reaction conditions: 5 mol% of 2-methoxyl-4-propylphenol in benzene, 350 °C, 90 bar, $H_2$ flow rate 30 mL/min, weight hourly space velocity (WHSV) 40 $h^{-1}$.

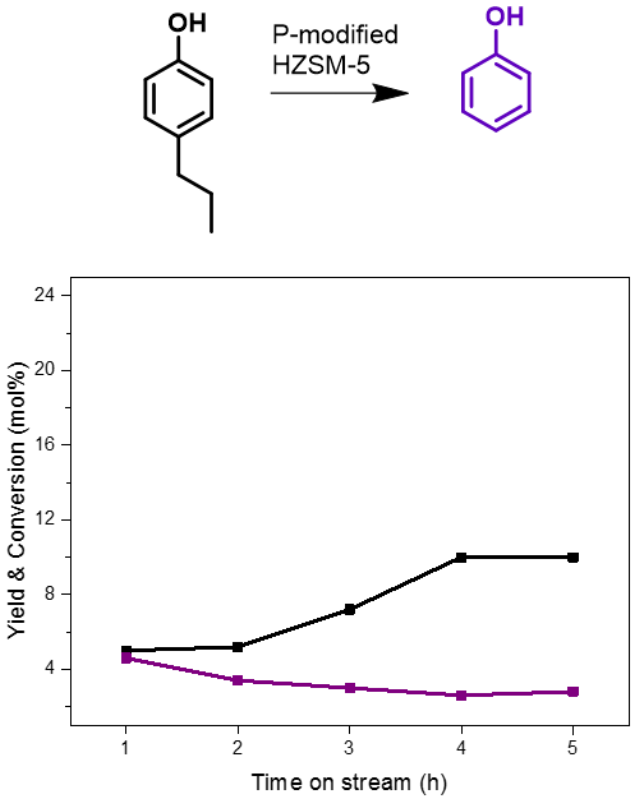


**Figure S8** Conversion of propylphenol to phenol using phosphate-modified HZSM-5. Reaction conditions: 100 mg phosphate modified HZSM-5, 0.15 mL/min 5 mol% of 4-propylphenol in benzene, 350 °C, 90 bar, gas flow rate 30 mL/min $H_2$.

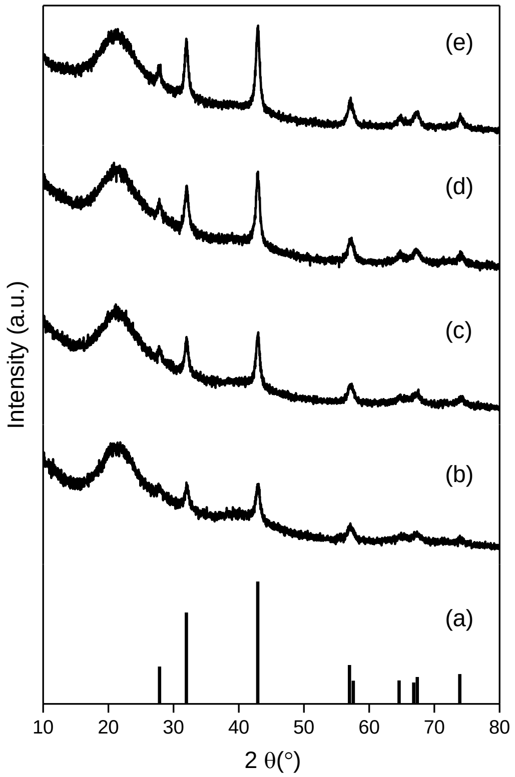


**Figure S9** XRD signals of (a) MoP/SiO2 in the PDF database and XRD patterns of MoP/$SiO_2$ catalysts obtained at different reduction temperatures: (b) 600 °C, (c) 700 °C, (d) 800 °C and (e) 900 °C. MoP formation was also confirmed by XRD with all diffraction lines belonging to MoP. From the sharpening of the diffraction lines, we can also conclude that the MoP particles became slightly larger when the reduction temperature was increased (see TEM in figure S9).

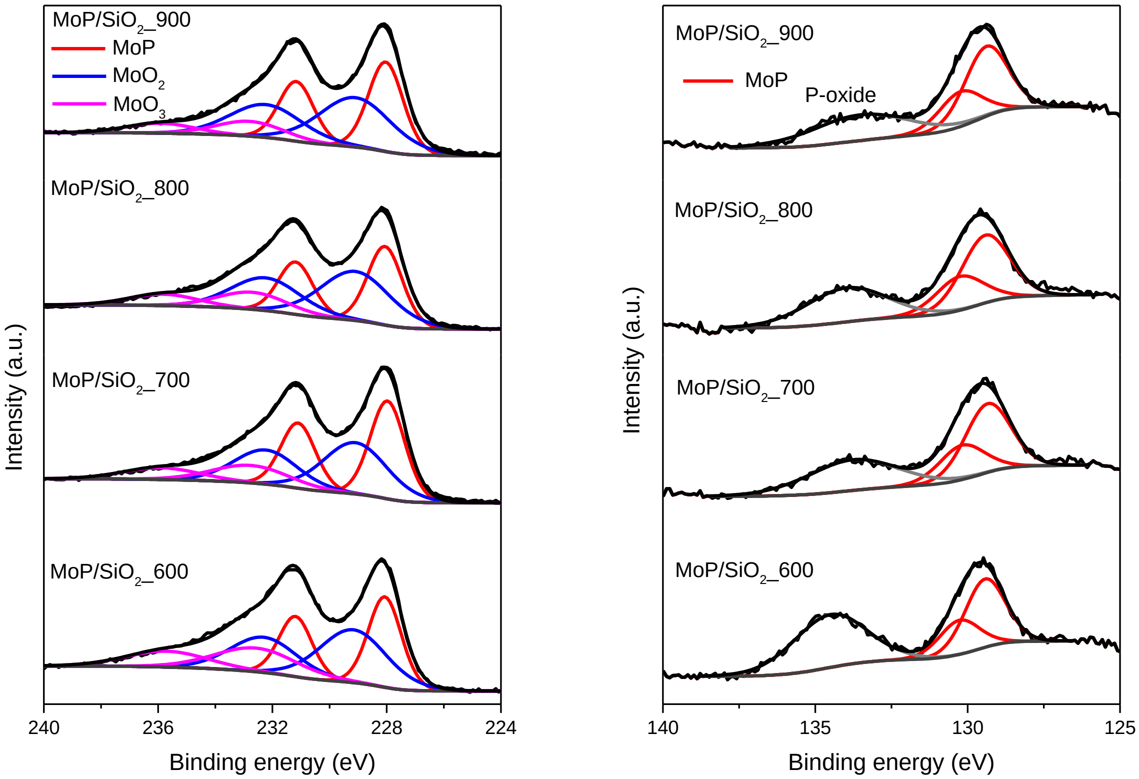


**Figure S10** XPS results of $MoP/SiO_2$ catalysts synthesized at different temperature (600 °C, 700 °C, 800 °C and 900 °C). Left figure: Mo 3d spectra; Right figure: P 2p spectra. Figure S8 (right) shows Mo 3d spectra of as-synthesized $MoP/SiO_2$ catalysts. Reduced Mo is characterized by the Mo $3d_{5/2}$ signal at 228.0 eV. The other features with binding energies of 229.2 eV and 232.8 eV can be assigned to $Mo^{4+}$ and $Mo^{6+}$ species, respectively. In Figure S8 (left), P 2p spectra show the presence of both phosphate and phosphide species. With increasing reduction temperature, phosphate is reduced to phosphide.

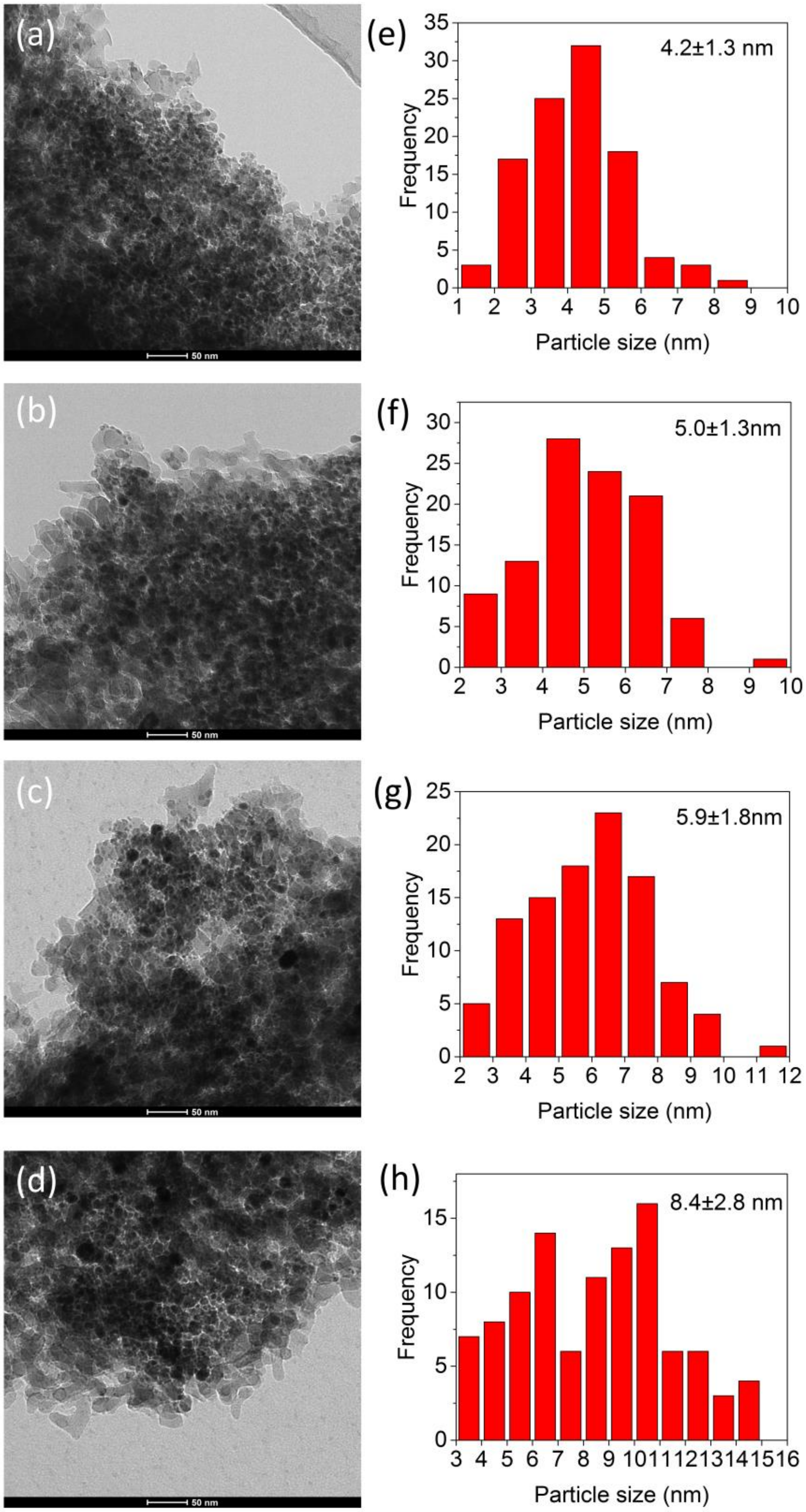


**Figure S11** TEM images and particle size distribution of $MoP/SiO_2$ catalysts reduced at different temperatures: (a, e) 600 °C, (b, f) 700 °C, (c, g) 800 °C, (d, h) 900 °C.

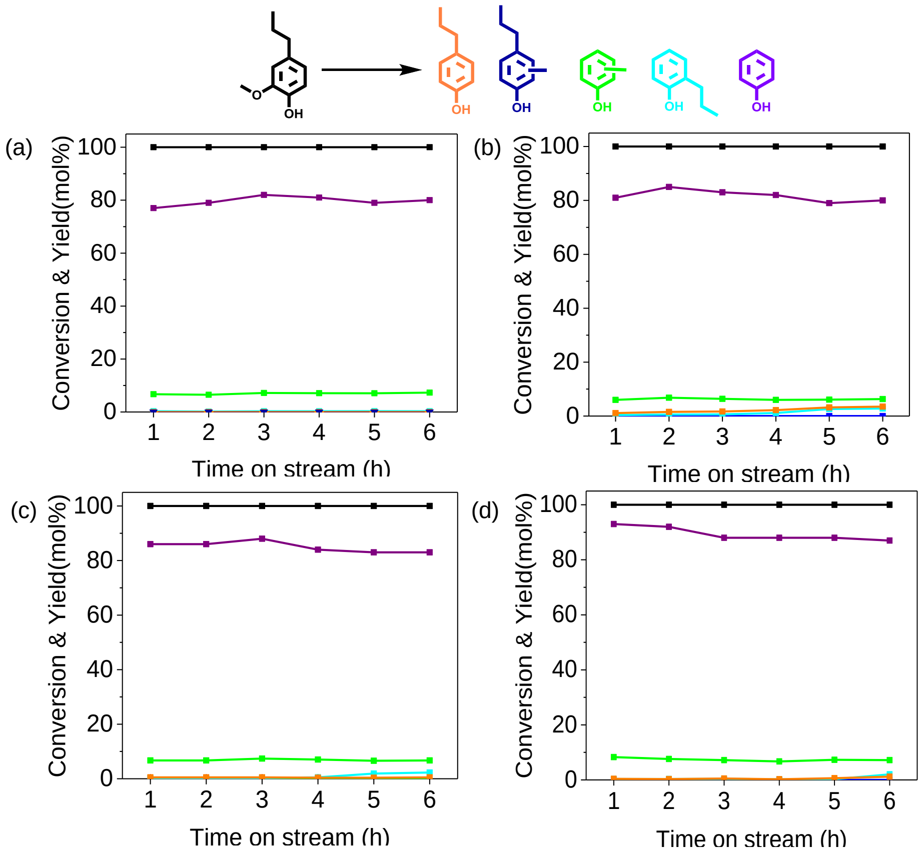


**Figure S12** One-step conversion of 2-methoxy-4-propylphenol into phenol in a fixed-bed flow reactor using HZSM-5 and MoP/$SiO_2$ synthesized at (a) 600 °C, (b) 700 °C, (c) 800 °C and (d) 900°C. Pretreatment conditions: the mixture of MoP/ $SiO_2$ and HZSM-5 is reduced in 100 mL/min $H_2$ at 450 °C for 1 hour. Reaction conditions: 5 mol% of 2-methoxyl-4-propylphenol in benzene, 350 °C, 90 bar, gas flow rate 30 mL/min $H_2$, weight hourly space velocity (WHSV) 40 $h^{-1}$.

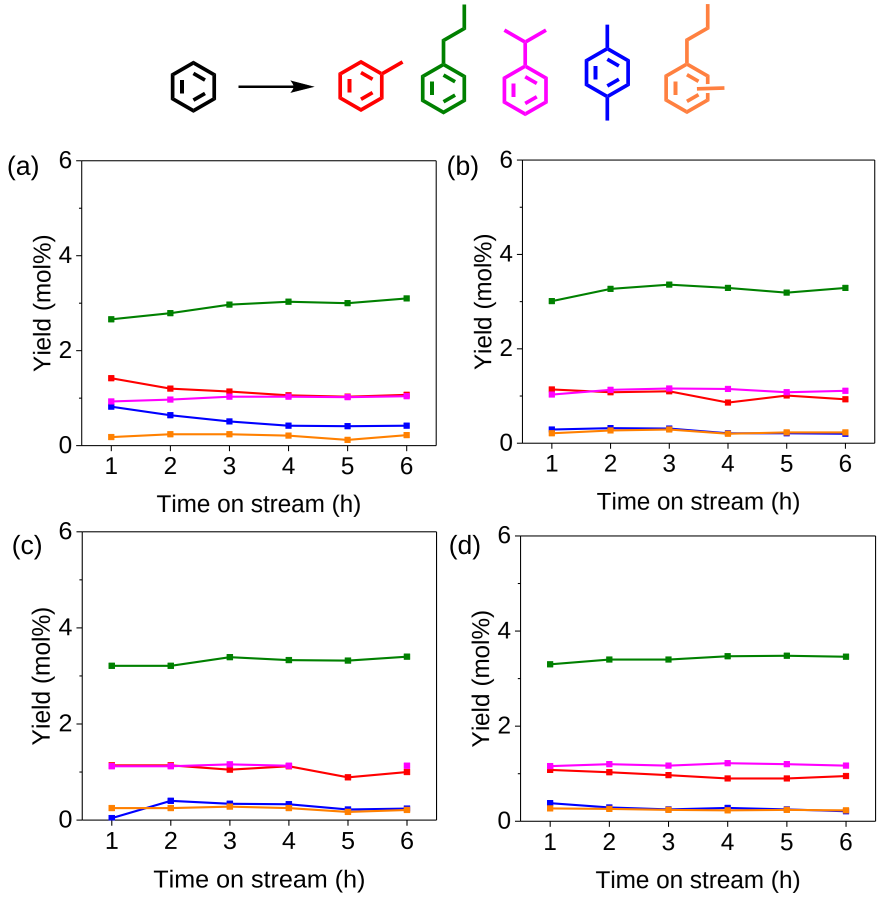


**Figure S13** The solvent-derived products in the phenol production from 2-methoxyl-4-propylphenol using the mixture of HZSM-5 catalyst and MoP/$SiO_2$ catalysts synthesized at different temperature (a) 600 °C, (b) 700 °C, (c) 800 °C and (d) 900 °C. Pretreatment conditions: 100 mg of catalyst is reduced in 100 mL/min $H_2$ at 450 °C for 1 hour. Reaction conditions: 5 mol% of 2-methoxyl-4-propylphenol in benzene, 350 °C, 90 bar, gas flow rate 30 mL/min $H_2$, weight hourly space velocity (WHSV) 40 $h^{-1}$.

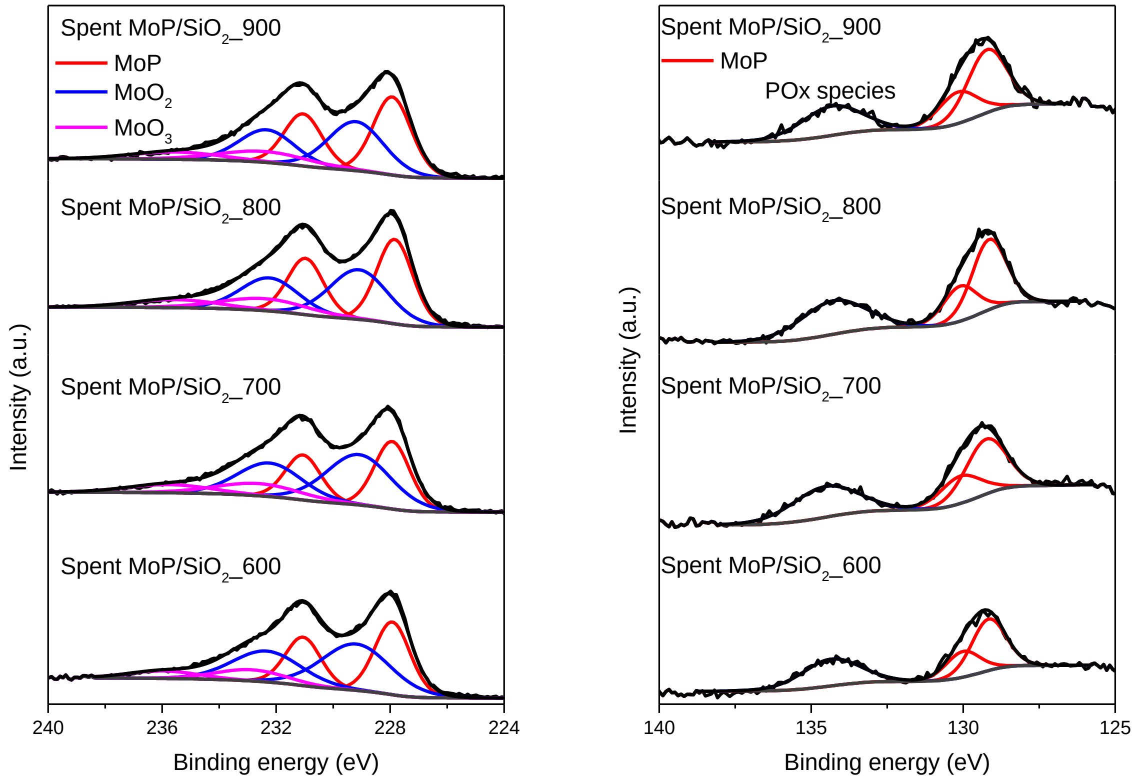


**Figure S14** XPS results of used MoP/$SiO_2$ catalysts synthesized at different temperature (600 °C, 700 °C, 800 °C and 900 °C). Left figure: Mo 3d spectra; Right figure: P 2p spectra.

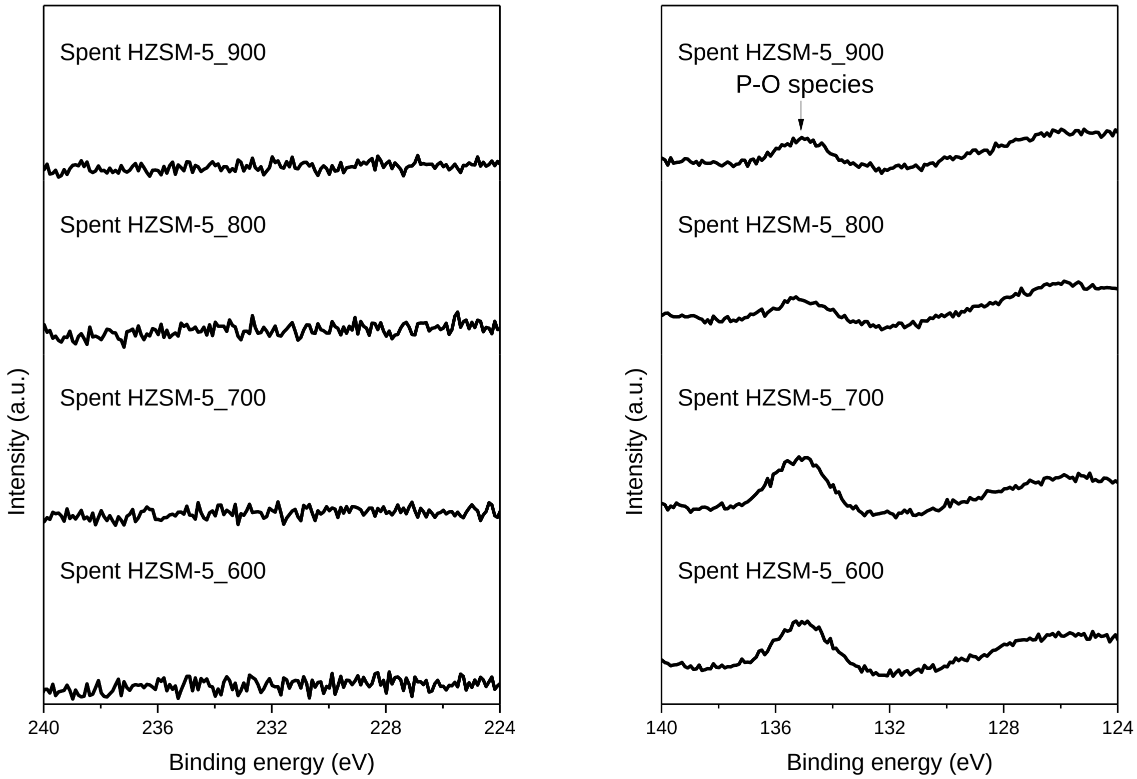


**Figure S15** XPS results of used HZSM-5 catalysts separated from the used $MoP/SiO_2$ synthesized at different temperature (600 °C, 700 °C, 800 °C and 900 °C). Left figure: Mo 3d spectra; Right figure: P 2p spectra.

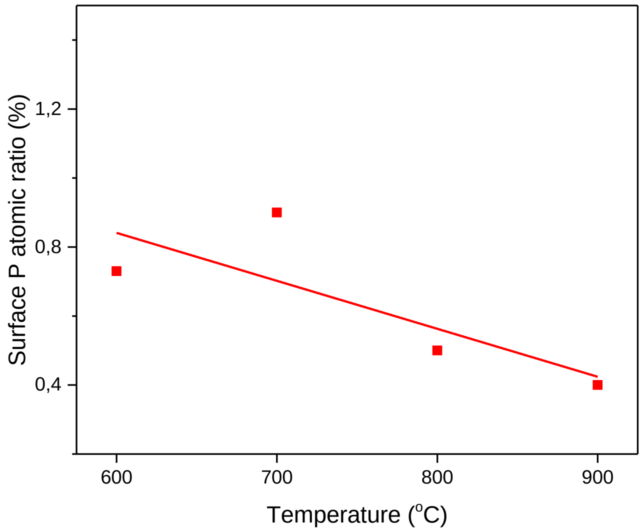


**Figure S16** Phosphorus content on the used HZSM-5 catalysts separated from the used MoP/$SiO_2$ synthesized at different temperature (600 °C, 700 °C, 800 °C and 900 °C). XPS survey spectra is used to calculate the surface phosphorus content.

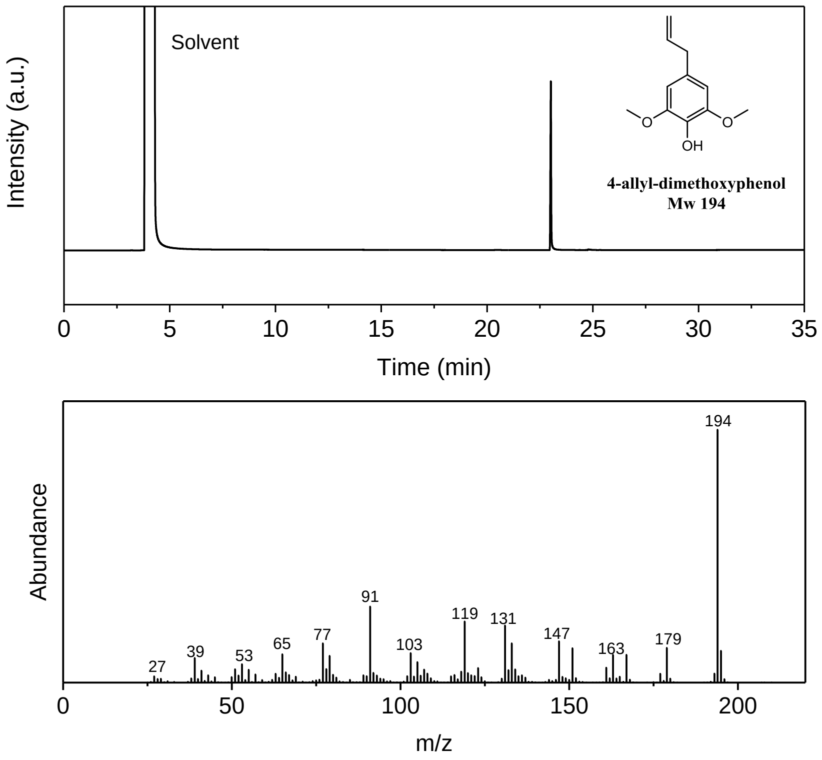


**Figure S17** GC-MS analysis of 4-allyl-dimethoxyphenol.

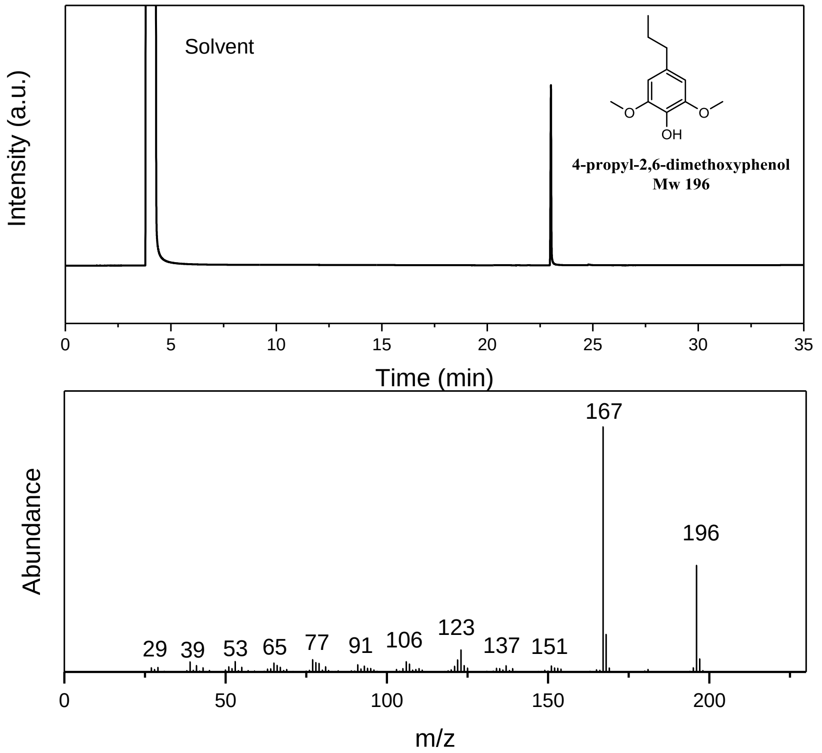


**Figure S18** GC-MS analysis of 4-propyl-2,6-dimethoxyphenol.

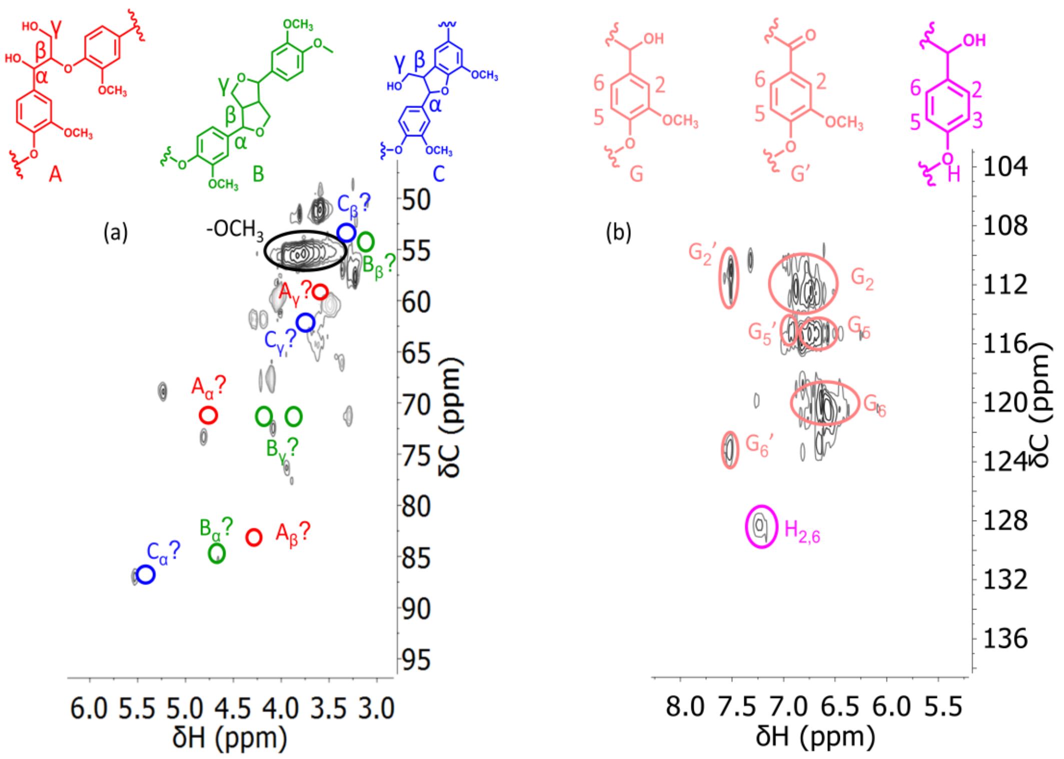


**Figure S19** Heteronuclear single quantum coherence NMR spectroscopy of obtained lignin oil after Pt/C catalyzed depolymerization.

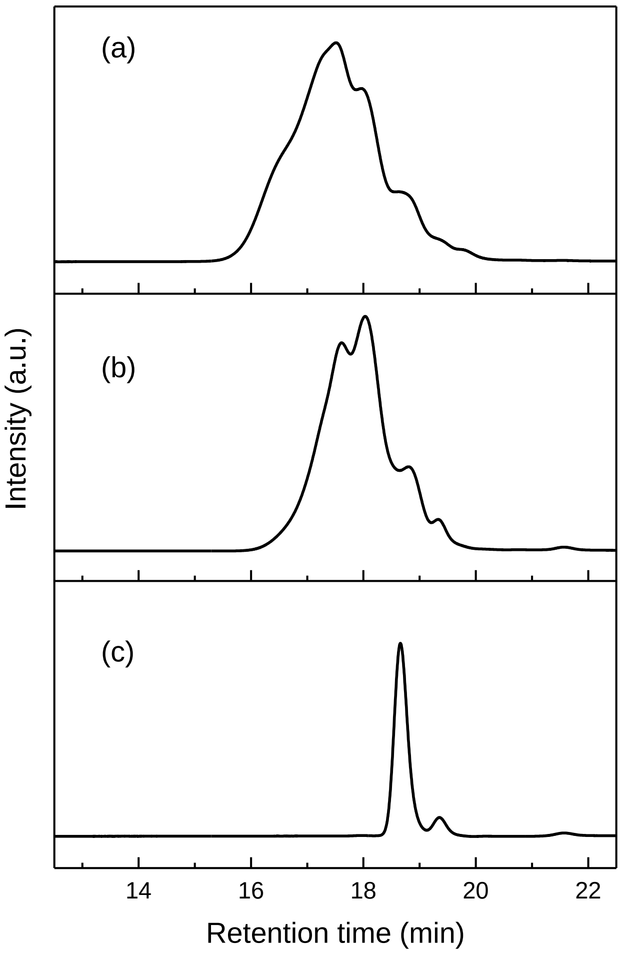


**Figure S20** Gel permeation chromatography of lignin oil after Pt/C catalyzed depolymerization (a), lignin oil extracted by benzene (b) and the 2-methoxy-4-propyl phenol model compound (c).

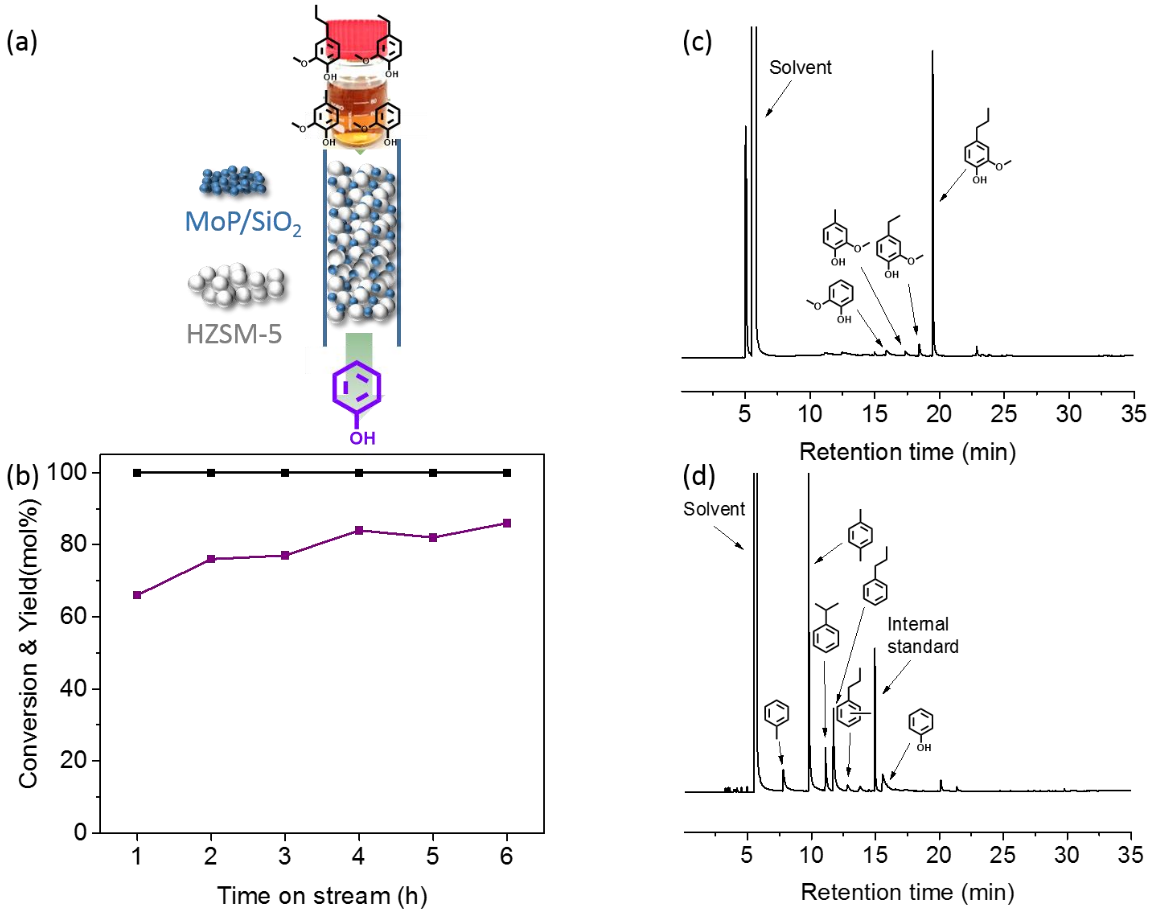


**Figure S21** Catalytic conversion of pinewood lignin oil to phenol (a), monomers conversion and phenol yield (b). Pretreatment conditions: a mixture of 100 mg $MoP/SiO_2$ and 100 mg HZSM-5 was reduced in 100 mL/min $H_2$ at 450 °C for 1 hour. Reaction conditions: 0.5 mol% of obtained lignin monomers in benzene, 0.1 mL/min, 350 °C, 90 bar, $H_2$ flow rate 30 mL/min $H_2$. Gas chromatograms highlighting the monomers obtained after Pt/C catalyzed pinewood LFP of pinewood in a batch reactor (c) and after the conversion of the lignin oil in a continuous-flow reactor using a mixed bed of HZSM-5 and $MoP/SiO_2$ (d).

## C. Tables

**Table S1** Chemical analysis and textural properties of silica supported transition metal phosphide catalysts.

| Catalyst | Metal loading (mmol/g support)[a] | $S_{BET}$ $(m^2/g)$[b] | CO uptake (umol/g) | Metal loading (mmol/g support)[c] | Phosphorus loading (mmol/g support)[c] |
|---|---|---|---|---|---|
| $SiO_2$ | - | 326 | - | | - |
| $Ni_2P/SiO_2$ | 1.6 | 219 | 28 | 1.5 | 0.9 |
| $CoP/SiO_2$ | 1.6 | 206 | 15 | 1.4 | 1.2 |
| $FeP/SiO_2$ | 1.6 | 110 | 10 | 1.3 | 1.5 |
| $WP/SiO_2$ | 1.6 | 154 | 13 | 1.3 | 1.2 |
| $MoP/SiO_2$ | 1.6 | 201 | 204 | 1.2 | 1.1 |

[a] Targeted metal loading 1.6 mmol/g support; [b] $S_{BET}$: BET surface area determined by $N_2$ physisorption; [c] Actual metal and phosphorus loading determined by ICP-OES analysis.

**Table S2** Melting point and loading of metals.

| Metal | Tungsten | Molybdenum | Cobalt | Nickel | Iron |
|---|---|---|---|---|---|
| Melting point ($^o$ C) | 3400 | 2620 | 1495 | 1453 | 1127-1593 |
| Metal loading (wt%) | 22.7 | 13.3 | 8.6 | 8.6 | 8.2 |

**Table S3** Catalytic results of 2-methoxy-4-propyl phenol conversion for 48 hours.[a]

| Reaction time (h) | Conversion(mol%) | Main product yield (mol%) | |
|---|---|---|---|
| | | Phenol | Propyl phenol |
| 1 | 100 | 89 | 1 |
| 3 | 100 | 87 | 2 |
| 6 | 100 | 81 | 5 |
| 9 | 100 | 72 | 11 |
| 25 | 100 | 56 | 22 |
| 48 | 100 | 9 | 70 |
| Catalyst regeneration[b] | - | - | - |
| 1[c] | 100 | 85 | 5 |

[a] 100 mg MoP/SiO2, 100 mg HZSM-5. Pretreatment conditions: the mixture of MoP/ $SiO_2$ and HZSM-5 is reduced in 100 mL/min $H_2$ at 450 °C for 1 hour. Reaction conditions: 5 mol% of 2-methoxyl-4-propylphenol in benzene, 350 °C, 90 bar, gas flow rate 30 mL/min $H_2$, weight hourly space velocity (WHSV) 40 $h^{-1}$. [b] Catalyst regeneration: 100 mL/min $H_2$, 700 $^o$ C, 2 h.[c] First-hour sample after the regeneration.

**Table S4** Results of catalytic HDMeO of 2,6-dimethoxy-4-propylphenol.[a]

| Catalyst | Conversion (mol%) | Product yield (mol%) | | | | | |
|---|---|---|---|---|---|---|---|
| | 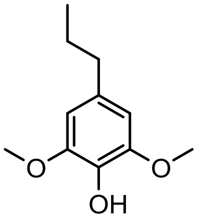 | 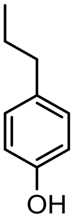 | 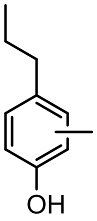 | 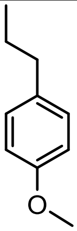 | 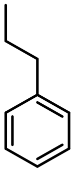 | 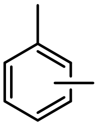 | 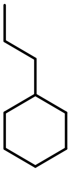 |
| $MoP/SiO_2$ | 100 | 23 | 8 | 3 | 3 | 1[b] | 2 |

[a] Reaction conditions: 1.0 g 2,6-dimethoxy-4-propylphenol, 100 mg $MoP/SiO_2$, 40 mL benzene, 50 bar of $H_2$ at room temperature, reaction temperature 350 °C, stirring 500 rpm, reaction time 2 h. [b] Xylene is probably formed by the alkylation between methoxyl groups and benzene solvent.

**Table S5** Catalytic transalkylation of 2,6-dimethoxy-4-propylphenol HDMeO products.[a]

| Catalyst | Conversion(mol%) | Product yield (mol%) | | Solvent-derived product yield (mol%) [b] | | |
|---|---|---|---|---|---|---|
| | | 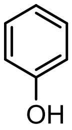 | 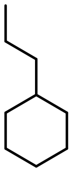 | 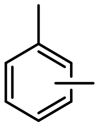 | 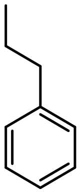 | 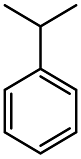 |
| HZSM-5 | 100 | 31 | 2 | 0.04 | 0.41 | 0.10 |

[a] Reaction conditions: products from HDMeO of 2,6-dimethoxy-4-propylphenol in Table S3, 100 mg HZSM-5, 50 bar of $H_2$ at room temperature, reaction temperature 350 °C, , stirring 500 rpm, reaction time 2 h.
[b] Solvent-derived product yield is based on the initial moles of benzene.
$$\text{Solvent derived product (mol\%)} = \text{moloes of solvent derived products/moloes of benzene} \times 100\%$$

**Table S6** Influence of reduction temperature on $MoP/SiO_2$ formation as analyzed by XPS

| Reduction temperature/°C | Mo species | | | P species | | Mo/P ratio [a] | $Mo^{\delta+}/P^{\delta-}$ ratio [b] |
|---|---|---|---|---|---|---|---|
| | $Mo^{\delta+}$ | $Mo^{4+}$ | $Mo^{6+}$ | $P^{\delta-}$ | P-oxides | | |
| 600 | 57 | 14 | 29 | 67 | 33 | 1.08 | 0.92 |
| 700 | 61 | 27 | 11 | 77 | 23 | 1.01 | 0.80 |
| 800 | 58 | 31 | 11 | 78 | 22 | 1.08 | 0.80 |
| 900 | 61 | 31 | 8 | 79 | 21 | 1.13 | 0.87 |

[a] The Mo/P ratio is calculated from the peak area of the Mo 3d region and P 2p region in the XPS survey spectra. $\mathrm{Mo/P\ ratio} = \frac{\mathrm{Mo\ region\ area/Mo\ RSF}}{\mathrm{P\ region\ area/P\ RSF}}$

[b] The Mo $^{\delta+}$/P $^{\delta-}$ ratio is calculated as follow: $\mathrm{Mo}^{\delta+}/\mathrm{P}^{\delta-} = \frac{\mathrm{Mo}^{\delta+}}{\mathrm{P}^{\delta-}} \times \mathrm{ratio}_{\mathrm{Mo/P}}$

## D. References


(1) A. Sluiter, B. Hames, R. Ruiz, C. Scarlata, J. Sluiter, D. Templeton and D. Croker, Determination of Structural Carbohydrates and Lignin in Biomass Determination of Structural Carbohydrates and Lignin in Biomass, National Renewable Energy Laboratory, **2008**.

(2) S. Van den Bosch, W. Schutyser, R. Vanholme, T. Driessen, S.-F. Koelewijn, T. Renders, B. De Meester, W. J. J. Huijgen, W. Dehaen, C. M. Courtin, B. Lagrain, W. Boerjan, B. F. Sels, *Energy Environ. Sci.* **2015**, *8* (6), 1748–1763.
